# Analysis of volatile organic compounds in Korean-bred strawberries: insights for improving fruit flavor

**DOI:** 10.3389/fpls.2024.1360050

**Published:** 2024-03-18

**Authors:** Eungu Jee, Eunsu Do, Chan Saem Gil, Seolah Kim, Sun Yi Lee, Seonghee Lee, Kang-Mo Ku

**Affiliations:** ^1^ Department of Plant Biotechnology, College of Life Sciences and Biotechnology, Korea University, Seoul, Republic of Korea; ^2^ Department of Horticulture, College of Industrial Science, Kongju National University, Yesan, Republic of Korea; ^3^ National Institute of Horticultural and Herbal Science, Rural Development Administration, Wanju, Republic of Korea; ^4^ Gulf Coast Research and Education Center, Horticultural Sciences Department, Institute of Food and Agricultural Science, University of Florida, Wimauma, FL, United States

**Keywords:** strawberry flavor, strawberry VOCs, GC-MS, mesifurane, γ-decalactone (PubChem CID: 12813), Korean strawberry, TD tube, strawberry cultivars

## Abstract

**Introduction:**

The strawberry industry in South Korea has witnessed a significant 65% growth over the past decade, surpassing other fruits and vegetables in production value. While sweetness and acidity are well-recognized flavor determinants, the role of volatile organic compounds (VOCs) in defining the desirable flavor profiles of strawberries is also crucial. However, existing research has predominantly concentrated on a limited range of commercial cultivars, neglecting the broader spectrum of strawberry varieties.

**Methods:**

This study embarked on developing a comprehensive VOC database for a diverse array of strawberry cultivars sourced both domestically and internationally. A total of 61 different strawberry cultivars from Korea (45), the USA (7), Japan (8), and France (1) were analyzed for their VOC content using Tenax TA Thermo Desorption tubes and Gas Chromatography-Mass Spectrometry (GC-MS). In addition to VOC profiling, heritability was assessed using one-way ANOVA to compare means among multiple groups, providing insights into the genetic basis of flavor differences.

**Results and discussion:**

The analysis identified 122 compounds categorized into esters, alcohols, terpenes, and lactones, with esters constituting the majority (46.5%) of total VOCs in Korean cultivars. ‘Arihyang’, ‘Sunnyberry’, and ‘Kingsberry’ exhibited the highest diversity of VOCs detected (97 types), whereas ‘Seolhong’ showed the highest overall concentration (57.5mg·kg^-1^ FW). Compared to the USA cultivars, which were abundant in γ-decalactone (a peach-like fruity aroma), most domestic cultivars lacked this compound. Notably, ‘Misohyang’ displayed a high γ-decalactone content, highlighting its potential as breeding germplasm to improve flavor in Korean strawberries. The findings underscore the importance of a comprehensive VOC analysis across different strawberry cultivars to understand flavor composition. The significant variation in VOC content among the cultivars examined opens avenues for targeted breeding strategies. By leveraging the distinct VOC profiles, particularly the presence of γ-decalactone, breeders can develop new strawberry varieties with enhanced flavor profiles, catering to consumer preferences for both domestic and international markets.

## 1 Introduction

Strawberry (*Fragaria* ×*ananassa* Duch.) is a worldwide cultivated crop with sweetness and favorable fragrance. Strawberry cultivation has been expanded in recent decades, demanding various cultivars that guarantee mass production, environmental adaptability, prolonged shelf-life, and disease resistance (Hardigan et al., 2018). However, studies for sensory quality and fruit flavor have been frequently overlooked (Aharoni et al., 2004). As a result, modern breeding methods seem to diminish the aroma intensity of strawberries, especially when compared to their wild-type species (Whitaker et al., 2011; Vandendriessche et al., 2013). Modern strawberry breeding programs are increasingly prioritizing the enhancement of sweetness and fruity aroma as key factors in the development of new cultivar (Carrasco et al., 2005). Therefore, it is important to dissect the compositions of strawberry aroma and biosynthetic pathways of VOCs.

Strawberries have a distinct aroma primarily influenced by esters, which account for 20% to 90% of all volatiles (Yan et al., 2018). However, other chemical classes also play an important role in shaping the overall flavor and scent of the fruit. Over 360 VOCs have been identified in cultivated strawberries, contributing various chemical classes such as acids, aldehydes, ketones, and more, collectively defining the taste profile in strawberry fruits (Zhang et al., 2010). While the other essential components like nonvolatile sugars and organic acids play a significant role for taste, certain specific VOCs such as γ-decalactone and mesifurane are particularly pivotal in determining the sensory quality of strawberries (Pyysalo et al., 1979; Larsen et al., 1992; Zorrilla-Fontanesi et al., 2012; Porter et al., 2023). Enhancing aromatic flavors in fruits could create a perception of increased sweetness, even when there is minimal difference in sugar content between highly flavored and non-flavored fruits (Fan et al., 2021a).

To breed highly flavored strawberry cultivars, an initial step involves determining favorable aromatic volatiles and quantifying their content in fruits from various genetic origins (Vandendriessche et al., 2013). Several studies have been reported for understanding the role of VOCs in strawberry fruits using various metabolomic methods. Odor Activity Values (OAVs) are commonly used to identify essential volatile compounds (Grosch, 1994; Nuzzi et al., 2008). In addition, to investigate the distinctive aroma of strawberries, sensory panel tests were applied and could identify key VOCs related to fruity flavor. Consequently, it is well known that several VOCs produce the unique scent in strawberry fruits by synergistically harmonizing each other. For example, one notable compound associated with sweetness is γ-decalactone, which contributes a peach-like aroma. Besides, caramel-like flavor can be attributed to furaneol and mesifurane, while floral-like fragrances commonly derived from linalool and nerolidol in strawberry fruits (Schieberle and Hofmann, 1997; Ulrich et al., 1997; Urrutia et al., 2017).

Recently, many efforts have been tried to understand the genetic information of strawberries with advanced technologies, such as Genome-Wide Association Study (GWAS) and Marker-Assisted Selection (MAS) (Chambers et al., 2014; Uffelmann et al., 2021). These techniques expedite the identification of optimal breeding lines, enhancing specific and selective traits within a shortened breeding cycle (Cockerton et al., 2021). The primary aims of the breeding were on identifying quantitative trait loci (QTL) governing various traits, including disease resistance, soluble solids content, weight, TA, and fruit shape. Moreover, it has been reported that genomic selection could contribute significant improvement for yield and biomass of fruits (Gezan et al., 2017). To identify the QTLs, DNA markers, such as simple sequence repeats (SSR) and single nucleotide polymorphism (SNP), have been used (Lim et al., 2017; Verma et al., 2017; Nagamatsu et al., 2021; Fan et al., 2022). In particular, several metabolic studies to targeting specific strawberry VOCs, such as mesifurane and γ-decalactone, have facilitated enhancing strawberry quality with improved aromatic profiles (Zorrilla-Fontanesi et al., 2012; Oh et al., 2021).

Environmental factors during cultivation, harvest time (Schwieterman et al., 2014), and post-harvest periods (Do et al., 2024) can lead to variations in the physicochemical properties of most VOCs, making consistent and quantitative measurement challenging. This inconsistency can lead to biases in selecting target VOCs. Moreover, various methods available for measuring VOCs might overestimate certain aromas, introducing another potential bias (Da Costa and Eri, 2004). Another difficulty is to characterize the complex metabolic processes of strawberry VOCs in aroma enhancement. Because the enhancement of specific VOCs may inadvertently disrupt other metabolic pathways, potentially resulting in an overall less desirable aroma profile. Furthermore, individual consumer preferences can vary regarding the perception of the same VOC, and it could be challenging to select and determine optimal concentrations for universally preferred VOCs. Despite these challenges, achieving a pleasant strawberry aroma is vital from both qualitative and commercial perspectives. Therefore, in this study, we conducted experiments to identify important VOCs in Korean strawberry breeding populations. With application of the techniques, we explored the characteristics of VOCs in strawberry fruits of 61 cultivars and conducted the comparative analysis among the cultivars based on the VOC profiles.

## 2 Materials and methods

### 2.1 Plant material

Fresh strawberry fruits of 55 cultivars and six breeding lines were used to compare the VOC profiles (**Table 1**).

**Table 1:** Origin and parentage of examined strawberry cultivars.

Number	Cultivar	Origin (Country, year)	Parentage
1	*Aiberry*	Japan, 1983	Unknown
2	*Akihime*	Japan, 1992	*Kunowase × Nyoho*
3	*Albion*	United States, 2004	*DiamanteⅹCal94.16-1*
4	*Amaou*	Japan, 2003	*Kurume 53 × 92-46*
5	*Arihyang*	South Korea, 2017	*Tochiotome × Seolhyang*
6	*Benihoppe*	Japan, 1999	*Akihime × Sachinoka*
7	*Camarosa*	United States, 1993	*DouglasⅹCal 85.218-605*
8	*Camiroreal*	United States, 1996	*Cal89.230-7 ⅹ Cal90.253-3*
9	*Chandler*	United States, 1979	*‘Douglas’(U.S. Plant Pat. No. 4,487)ⅹ’C55’(Cal 72.361-105)*
10	*Chodong*	South korea, 1986	*Harunoka × Yachio*
11	*Daeun*	South Korea, 2009	*Akihime × Redpearl*
12	*Daewang*	South Korea, 2010	*Maehyang × Wongyo3111ho*
13	*Dahong*	South Korea, 2007	*Sachinoka × Maehyang*
14	*Damhyang*	South Korea, 2012	*Akihime × Maehyang*
15	*Danmi*	South Korea, 2007	*Maehyang × Amaou*
16	*Darselect*	France, 1998	*Parker ⅹ Elsanta*
17	*Diamante*	United States, 1997	*Cal 87.112-6 ⅹ Cal 88.270-1*
18	*Toyonoka*	Japan, 1984	*Himiko × Harunoka*
19	*Durihyang*	South Korea, 2017	*Sukhyang × Maehyang*
20	*Eunhyang*	South Korea, 2021	*Daewang × Durihyang*
21	*Festival*	United States, 2000	*Rosa LindaⅹOso Grande*
22	*Gamhong*	South Korea, 2009	*Johong × Maehyang*
23	*Gaviota*	United States, 1992	*Cal 87.112-6 ⅹ Cal 88.270-1*
24	*Highberry*	South Korea, 2018	*NS071608 × Daewang*
25	*Jinmi*	South Korea, 2007	*Sachinoka × Amaou*
26	*Johong*	South Korea, 2002	*Nyoho × Akihime*
27	*Jukhyang*	South Korea, 2009	*Maehyang × Seolhyang*
28	*Kingsberry*	South Korea, 2016	*Akihime × NS001309*
29	*Kuemhyang*	South Korea, 2005	*Akihime × NS970016*
30	*Kuemsil*	South Korea, 2016	*Maehyang × Seolhyang*
31	*Maehyang*	South Korea, 2001	*Tochinomine × Akihime*
32	*Manhyang*	South Korea, 2003	*Nyoho × Akanekko*
33	*Merryqueen*	South Korea, 2017	*Seolhyang × Maehyang*
34	*Mihong*	South Korea, 1996	*Toyonoka × Reiko*
35	*Misohyang*	South Korea, 2017	*Darselect × Wongyo3111ho*
36	*NS160802*	South Korea, 2016	Unknown
37	*NS170310*	South Korea, 2017	*J090502 × Vitaberry*
38	*NS170319*	South Korea, 2017	Unknown
39	*NS181702*	South Korea, 2018	*Arihyang × Portola*
40	*NS193008*	South Korea, 2019	Unknown
41	*NS211504*	South Korea, 2021	*Kuemsil × NS181702*
42	*Okmae*	South Korea, 2010	*Toyonoka × Maehyang*
43	*Redpearl*	Japan, 1993	*Aiberry × Toyonoka*
44	*Redstar*	South Korea, 2021	*Benihoppe offspring*
45	*Sachinoka*	Japan, 1996	*Toyonoka × Aiberry*
46	*Seeberry*	South Korea, 2013	*Wongyo3115ho × Wongyo3116ho*
47	*Seolhong*	South Korea, 1994	*Suhong × Toyonoka*
48	*Seolhyang*	South Korea, 2005	*Akihime × Redpearl*
49	*Sinseolmae*	South Korea, 2012	Unknown
50	*Ssanta*	South Korea, 2009	*Maehyang × Seolhyang*
51	*Suhong*	South Korea, 1985	*Hokowase × Harunoka*
52	*Suhyang*	South Korea, 2009	*kuemsil × Highberry*
53	*Sukhyang*	South Korea, 2009	*Akihime × Redpearl*
54	*Sukyung*	South Korea, 2008	*Johong × Maehyang*
55	*Sunhong*	South Korea, 2005	*Johong × Maehyang*
56	*Sunnyberry*	South Korea, 2017	*Kaho × Reiko*
57	*Sunshine*	South Korea, 2021	*Benihoppe × Vitaberry*
58	*Tochiotome*	Japan, 1996	*Kurume-49 × Tochinomine*
59	*Vitaberry*	South Korea, 2014	*NS001309 × Daewang*
60	*Wongyo3111ho*	South Korea, 2008	*Benihoppe offspring*
61	*Wongyo3114ho*	South Korea, 2010	*Benihoppe offspring*

The fresh fruits were obtained from 20 plants of each cultivar and breeding line during winter season in 2022 and 2023. The plants cultivation and fruit harvest were conducted at the National Institute of Horticultural and Herbal Science (35°50′02.0*''*N 127°01′60.0*''*E, Wanjugun, Korea) under controlled conditions. During cultivation time, the plants were grown on a commercial substrate comprised of cocopeat and perlite (Chamgeuro Industrial Co., Hongweong-gun, Korea) through hydroponic systems using strawberry-specific nutrient solution. The solution was provided to the plants every two times per day with 30 mL at 10:00 a.m. and 04:00 p.m. via an automated drip system, ensuring optimal hydration and nutrient delivery. The utilized nutrient solution maintained an electrical conductivity (EC) of 0.4–0.5 dS·m^-1^ and a pH range between 5.5 and 5.7. The solution composition encompassed several macro and micro elements: NO_3_-N at 6.0 me·L^-1^, NH_4_-N at 0.2 me·L^-1^, PO_4_-P at 2.0 me·L^-1^, K at 3.5 me·L^-1^, Ca at 3.0 me·L^-1^, Mg at me·L^-1^, SO_4_-S at 1.0 me·L^-1^, Fe at 1.3 me·L^-1^, B at 0.3 me·L^-1^, Mn at 0.3 me·L^-1^, Zn at 0.3 me·L^-1^, Cu at 0.02 me·L^-1^, and Mo at 0.01 me·L^-1^. The greenhouse conditions were meticulously managed to accommodate the daily temperature fluctuations, maintaining temperatures above 7°C at night and below 25°C during the day, to support optimal plant growth.

### 2.2 Collecting untargeted VOCs from strawberry fruits

During the harvest period in January and February 2023, for over three replicates, more than 12 fruits were collected from each cultivar and breeding line between 09:00 and 11:00 a.m. when the temperature had not yet increased in the greenhouse. The fruits exhibited 90% maturity, a stage where about 90% of the fruit is colored, indicating commercial ripeness. VOCs were collected in thermal desorption (TD) tubes (Tenax TA Cond/Capped Pk 10, C1-AAXX-5003, Markes International, CA, USA) using the headspace method. Strawberries weighing 100 ± 5g were thoroughly mashed by hand and then placed inside a 15 × 20 mm polyethylene bag, made of non-reactive polyethylene, before being placed in a Magenta box (polycarbonate, W75 × D75 × H100 mm). The bag was then placed inside a Magenta box (polycarbonate, W75 × D75 × H100 mm). The top of the box was sealed with plastic wrap, and a lid with holes was securely placed on top. The samples were then saturated in an incubator at 30°C for 20 minutes. Volatile compounds were captured at a flow rate of 0.15 L min^−1^ for 2 minutes by connecting the TD tube to plastic tubing (Tygon S3 E-3603, Tygon S3™, Ohio, USA) connected to a pump (Mini pump MP-∑30KNII, Taewon SIBATA Co., Korea).

### 2.3 VOCs analysis by GC–MS

3-Octanone (Sigma-Aldrich Co., St. Louis, MO, USA) was used as an internal standard (IS). After capturing the VOCs, 3-Octanone was dissolved in methanol to a concentration of 25 μg/mL. Using an injection syringe, 4 μL of this solution was injected into the tube. Subsequently, the solvent was evaporated using a Solution Loading Rig (Markes International, California, USA) to prepare the sample for analysis.

The sample tubes were loaded into the TD-100-xr thermal desorption unit (Markes International, California, USA) for analysis. The desorption of samples was performed according to the following settings: Pre-desorption: Pre-purge time: 1 min, Split flow: 20 mL/min; Primary tube desorption: 240°C for 5 min with Trap flow: 40 mL/min. The sample tube was desorbed entirely (splitless desorption) onto the cold trap (U-T11GPC-2S, Markes International, California, USA) held at 25°C.

The cold trap was then heated at a rate of 25°C/s to 270°C and held for 5 minutes. During trap desorption, samples were split using a 6:1 outlet split. Analysis was conducted using a gas chromatograph-mass spectrometer (GCMS-QP 2020 NX, Shimadzu, Japan) equipped with a DB-5MS capillary column (Agilent Technologies, 30 m × 0.25 mm, 0.25 μm). The GC oven was initially held at 35°C for 3 minutes, then increased at a rate of 6°C/min to 170°C, followed by a steady increase at 12°C/min to 280°C. The total run time was 34 minutes. The MS ion source and interface temperatures were both 250°C, with ions acquired in the m/z range of 40-400. Peaks in the GC-MS chromatogram was integrated using Shimadzu GCMS solution software and compared against the NIST17 library for identification. Peak deconvolution was performed with Automated Mass spectral Deconvolution and Identification System (AMDIS) software (version 2.73, NIST), using specific parameters for accuracy. The AMDIS settings included: Minimum match factor: 75; Multiple identifications per compound, show standards, only reverse search: unchecked; Type of analysis: simple; Low m/z: Auto; High m/z: Auto; Threshold: High; Scan direction: None; Instrument type: Quadrupole; Deconvolution – 12 Component width; Adjacent peak subtraction: None; Resolution: High; Sensitivity: Low; Shape requirements: Medium; Target Compounds Library; Solvent tailing 84m/z; Column bleed 207 m/z; Scan Sets: Number of sets: 0, End 9999, Low m/z 0, High m/z 0; No use of Filter. Kovats indices were determined using a C7 to C21 alkane range. For compounds lacking standard samples, identification relied on the NIST Library with a minimum match factor of 75.

### 2.4 Statistical analysis method

#### 2.4.1 VOCs identification and quantification

For the VOCs Identification and Quantification process, IBM SPSS statistics 27 software (IBM Corp, Armonk, NY, USA) was used. Data normality was assessed using the Shapiro-Wilk test, and in cases where data did not exhibit a normal distribution, non-parametric tests were applied. The test for homogeneity of variances followed the Levene’s test. When addressing non-parametric ANOVA, the Kruskal-Wallis test was used. Specifically, absolute quantity data indicated that concentration of VOCs in the alcohol class followed a normal distribution, whereas other data did not. For *post-hoc* tests in parametric ANOVA, the Tukey HSD method was utilized.

Additionally, alkanes constituted a significant portion of the total VOCs, they, along with compounds from other classes suspected to be contaminants, were excluded from the analysis when classifying the total VOCs in strawberry cultivars by their functional groups. This exclusion is due to alkanes being predominantly odorless and therefore having minimal impact on the overall aroma profile (Zviely, 2004; Ouellette and Rawn, 2015). Additionally, compounds from other classes were also removed due to suspicions of them being contaminants.

#### 2.4.2 Hierarchical clustering analysis and concentration comparison

Hierarchical Clustering Analysis (HCA) and comparison of concentrations through the Box & Whisker plot were conducted using the Origin Pro Software (Origin Lab Corporation, Northampton, MA, USA), version 2023b. To facilitate Hierarchical Clustering Analysis (HCA), we first calculated the average values for each replicate of the cultivars and then standardized the data across all compounds. For HCA, the cluster method employed was ‘Group average’ and the distance type was ‘Euclidean’. The clustering was visualized in a circular shape using the ‘Find Clustroid by sum of distances’ method.

#### 2.4.3 One-way ANOVA analysis for heritability

A statistical software program that is free and open-source was used to conduct the analysis. The means between two groups were compared using the Student’s t-test, while the one-way ANOVA was employed to compare means among multiple groups. Tukey’s HSD test was then used as a *post-hoc* test in the ANOVA to determine p-values for multiple pairwise comparisons.

#### 2.4.4 PCA and heatmap analysis

MetaboAnalyst 6.0, an online tool for metabolomics data analysis, was used to analyze the data. The data was normalized using the median method and no transformations were applied. Auto scaling was employed for data scaling. Heatmap analysis of the data utilized Euclidean distance measure and ward clustering method with group average method for clustering purposes. Additionally, the French cultivar ‘Darselect’, a crossbreed involving the US cultivar ‘Parker’, was categorized with USA cultivars for ease of comparative analysis.

## 3 Results

### 3.1 VOCs identification and quantification

A comprehensive analysis was conducted on a total of 61 strawberry cultivars, both domestic and international. The samples were collected using Tenax TA tubes and analyzed through GC-MS, which allowed for the identification of 122 different VOCs. Detailed information regarding these compounds was referred to **Supplementary Table S1**.

**Figure 1:**
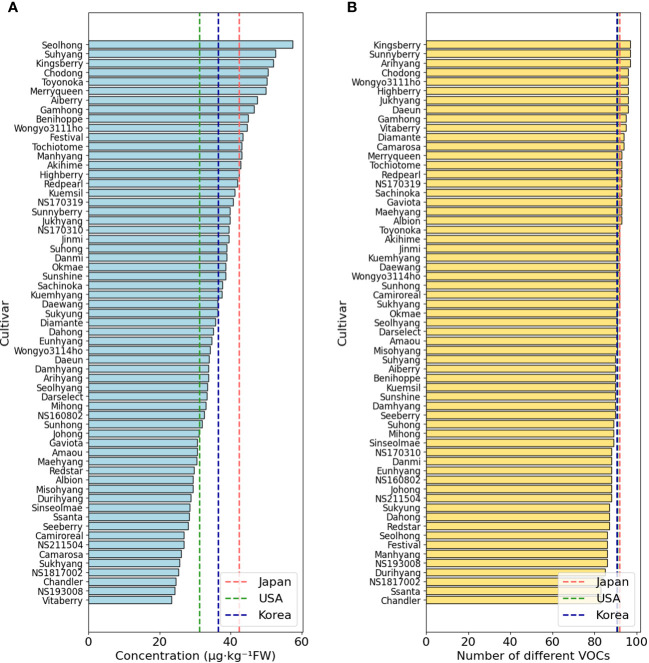
Comparison of VOC concentrations **(A)** and the number of different VOCs **(B)** across cultivars. Dashed lines mean average value for each country.

Among the cultivars or lines according to the origin, Japanese cultivars showed the highest amount of the total VOC content per a strawberry fruit, exhibiting 42.4 μg·kg^-1^ FW. The total amount was followed by samples from Korea at 36.5 μg·kg^-1^ FW, and samples from the United States at the lowest, with 31.3 μg·kg^-1^FW (**Figure 1A**). However, the average number of VOC compounds per strawberry cultivar showed different patterns, which exhibited that Korean samples had 90.7 compounds, Japanese samples had 91.8 compounds, and the United States samples had 90.8 compounds (**Figure 1B**).

In case of Korean samples, ‘Seolhong’ showed the highest amount of VOCs, which exhibited 57.5 μg·kg^-1^FW, while ‘Vitaberry’ contained the lowest amount of VOCs (23.4 μg·kg^-1^ FW). In terms of VOC diversity, ‘Arihyang’, ‘Sunnyberry’, and ‘Kingsberry’ exhibited the highest diversity as 97 compounds, whereas ‘Chandler’ and ‘Ssanta’ had the lowest diversity in VOCs as 83 compounds.

The coefficient of variance for VOC concentrations across different cultivars was 21.90, while the coefficient for the number of VOCs per cultivar revealed 3.91. Therefore, the result indicates that while the number of VOCs in each cultivar remains relatively constant, the concentration of these compounds exhibits more substantial variability, underscoring that it’s the concentration levels of VOCs that provide a more distinctive differentiation among cultivars than the number of VOCs present.

**Figure 2:**
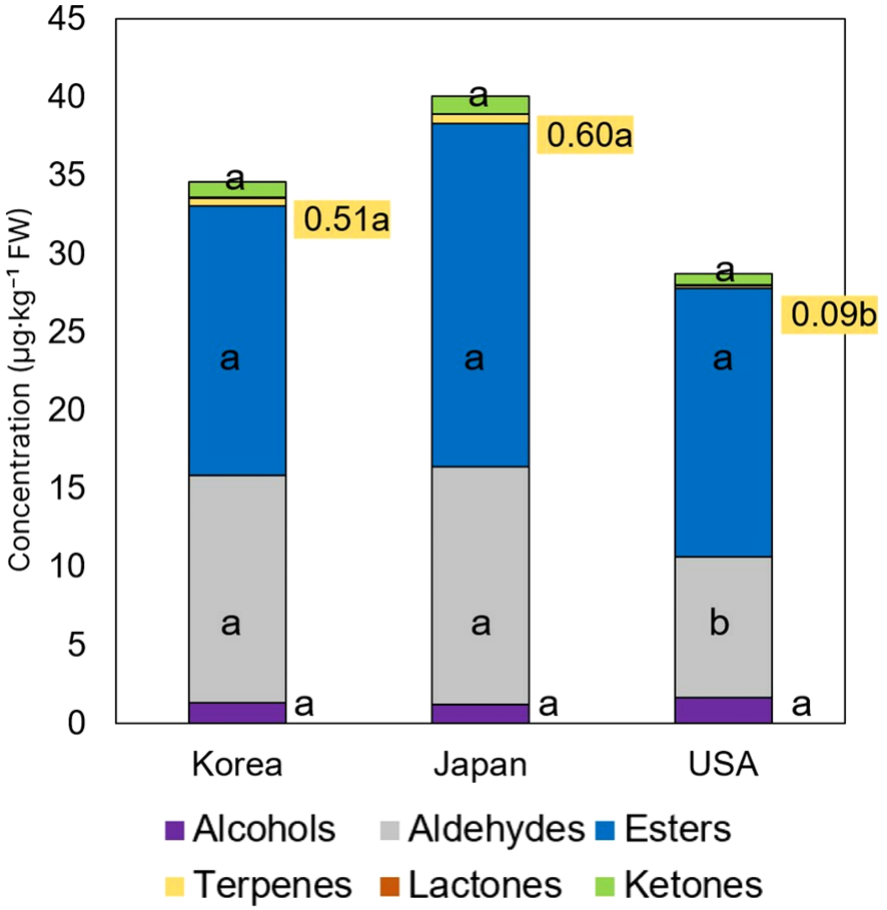
Comparison of major VOC content by class in strawberry cultivars from Korea, Japan, and USA. Different letters indicate significant differences in the content of each VOC class among the countries (*p* < 0.05).

**Figure 2** presents a classification of the total VOCs in strawberry cultivars by their functional groups, comparing the differences in their contents among cultivars from Korea, Japan, and the USA. The graph illustrates statistical differences in the quantities of each VOC class, denoted by distinct letters. The graph revealed that, generally, the quantities of compounds in Korean and Japanese cultivars showed similar tendency. Notably, the average levels of both aldehydes and terpenes were found to be significantly lower in USA cultivars. In Korean cultivars, the concentrations of alcohols, esters, and lactones were lower than those in USA cultivars, while ketones were slightly higher, though these differences were not statistically significant.

Additionally, we compared the proportions of VOCs according to the cultivar. Notably, in USA cultivars, where the concentrations of aldehydes and terpenes were lower, the proportion of alcohols was significantly higher, at 5.3%, compared to those from Japan and Korea, which had 2.9% and 3.6%, respectively. Upon calculating the average across the three countries, esters comprised about 50.8%, aldehydes 34.5%, alcohols 3.9%, ketones 2.6%, terpenes 1.2%, and lactones 0.17%. In contrast, the proportion of Esters in Korean cultivars was 46.5%, which is lower than the average. This indicates that Korean cultivars are deficient in these VOC classes, suggesting potential target compounds for flavor enhancement. The results indicate that while the total number of VOCs remains relatively consistent across the cultivars from the USA, Japan, and Korea, significant disparities are observed in their composition.

### 3.2 Principal component analysis and hierarchical clustering analysis

To examine and distinguish the traits among different cultivars, Principal Component Analysis (PCA) was implemented to analyze the VOCs data. PCA revealed that PC1 and PC2 accounted for 18.6% of the overall variance (PC1:11.5%, PC2:7.1%). Except for a few exceptional cases, majority of the samples exhibited overlapping patterns within the 95% confidence interval, indicating that PCA did not effectively differentiate between cultivars based on total VOC concentration (**Supplementary Figure S1**).

**Figure 3:**
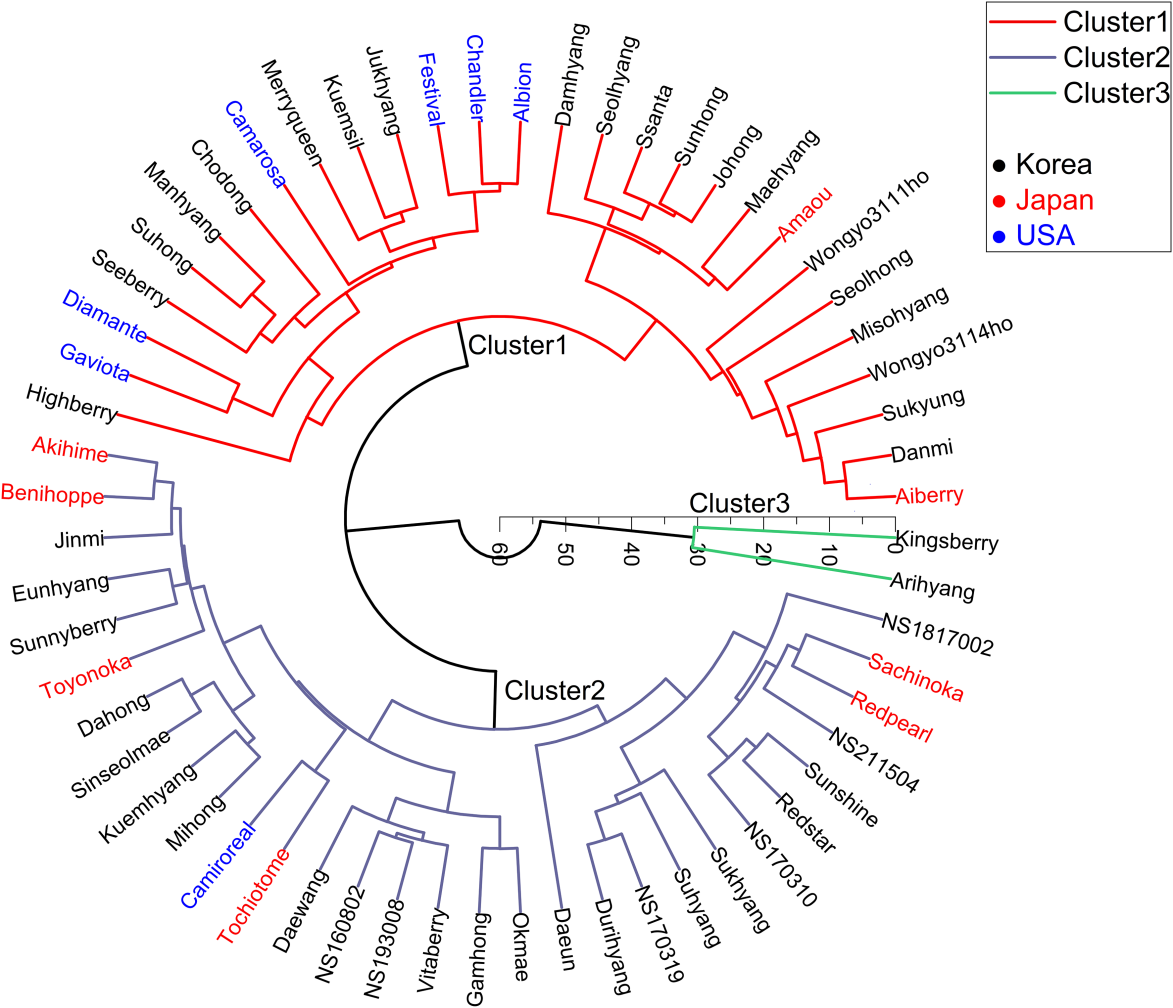
Hierarchical clustering analyses (HCA) of VOCs across cultivars categorized by country. The displayed VOC values for each cultivar are based on the average of three replicates. The Euclidean distance type was employed for clustering, and the number of clusters was set to 3.

To gain a preliminary understanding of the relationships among the 61 cultivars, Hierarchical Clustering Analysis (HCA) was performed on the entire VOCs composition of each cultivar (**Figure 3**). When the number of clusters was set to three and HCA was executed, 59 out of the 61 cultivars were split into two clusters, with the exceptions being ‘Kingsberry’ and ‘Arihyang’ (both in cluster 3). The column in the HCA diagram illustrates the dissimilarity or distance between clusters merged at each step. As the clustering process advances, the distance value escalates, suggesting that less similar clusters are being combined as the stages progress. While USA cultivars like ‘Camarosa’, ‘Diamante’, and ‘Gaviota’, and Japanese cultivars such as ‘Akihime’ and ‘Benihoppe’ tended to cluster closely, the VOC data alone did not reveal intuitive differences among Korean, Japanese, USA cultivars. This lack of differentiation is postulated to arise from the fact that the concentration of each VOC is more influenced by environmental factors associated with cultivation conditions than by genetic factors specific to each cultivar.

### 3.3 Assessing the genetic influence on strawberry VOCs

**Figure 4:**
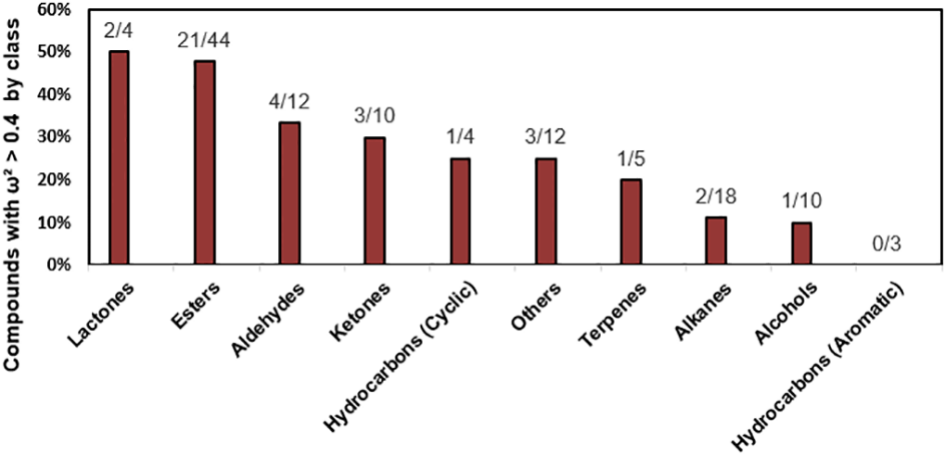
Percentage distribution of VOCs by compound class influenced by heritability. The graph represents the percentage of VOCs with a ω² value greater than 0.4 for each compound category. The fractions in parentheses indicate the number of VOCs with a ω² value exceeding 0.4 out of the total number of VOCs in that category.

Responding to changes of environmental conditions during cultivation, the accumulation of specific VOCs in strawberry fruits might be influenced. To dissect the variance caused by the genotype effect (G) regarding VOC accumulation, we divided the factors into genetic factors (G) and other factors. Using One-Way ANOVA, each factor or trait was calculated and quantified. The ω²value can serve as an estimate of heritability, indicating the proportion of genetic variance in the total variance. Following the method used by Pol Rey-Serra et al (Rey-Serra et al., 2022), we utilized the ω²value as an approximation of heritability. The analysis of variance (ANOVA), considering both the genotype and other factors, encompassed all 122 VOCs. Significance was determined at *p* < 0.05 level, resulting in the identification of VOCs that exhibited statistically significant differences. Out of the total 122 VOCs, approximately 31.1% exhibited a ω² greater than 0.4. Notably, substances with a ω² value exceeding 0.7, including methyl isovalerate, methyl isobutyl ketone, hexanoic acid, methyl ester, linalool, 2-Nonanol, and Acetate, were identified as compounds significantly influenced by heritability. When categorizing the results by compound class, lactones emerged as the most prominent, accounting for 50% (**Figure 4**), followed by esters at 48%. In contrast, compounds from classes such as alkane and alcohol exhibited significantly lower percentages. The higher proportions of lactones and esters can be attributed to their greater heritability compared to environmental factors. Therefore, these compound classes could be considered primary targets for breeding purposes. In summary, our study focused on 38 notable volatile organic compounds out of a larger pool of 122 VOCs. These selected VOCs were subjected to further analysis and examination. This additional analysis aimed to elucidate the distinctions among group means and identify particular groups that exhibit significant differences from one another (**Supplementary Table S1**).

### 3.4 Heatmap analysis of key volatile compounds across strawberry cultivars

Based on previous research, 19 volatile organic compounds were identified as that exhibits a strong correlation with the distinctive aroma of strawberries. These compounds are commonly referred to as the key volatile components present in wild strawberries and play a crucial role in determining their unique scent. Of particular significance is their ability to surpass the threshold at which humans can detect odors, and when combined, these 19 compounds create the characteristic aroma associated with strawberries (Urrutia et al., 2017).

**Figure 5:**
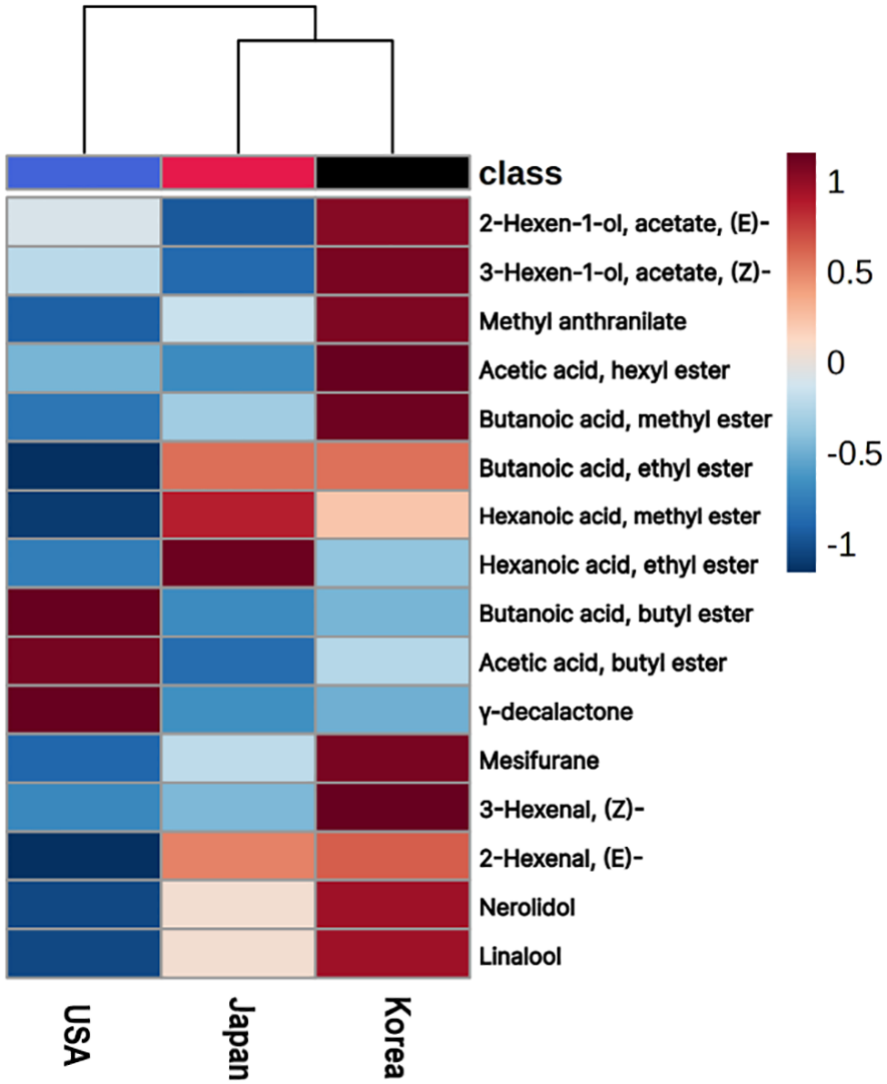
Heatmap of KVC in Korean, Japanese, and USA Strawberry Cultivars. The color scale bar on the right side represents the normalized value of compound concentrations from -1 to 1. The hierarchical clustering on the top indicates the similarity in VOC profiles among the three countries.

Adopting these insights, we conducted Gas Chromatography-Mass Spectrometry (GC-MS) analyses to identify the spectrum of VOCs in our samples. Out of the 122 types of VOCs identified, we specifically focused on the 19 KVCs, with 16 of these KVCs notably detected in our samples. Based on these findings, a heatmap was developed to effectively illustrate the variations in VOC concentrations, highlighting the differing levels of key volatile compounds found in strawberry cultivars from Korea, Japan, and the USA (**Figure 5**). Among the examined compounds, Korean cultivars exhibited significant levels of five out of 10 ester compounds, including 2-hexen-1-ol, acetate. They also demonstrated abundant amount of mesifurane classified in lactone group, aldehyde compounds like 2-hexenal, and terpene compounds such as linalool compared to cultivars from other countries. In contrast, Japanese cultivars showed higher concentrations of hexanoic acid in both its methyl and ethyl ester forms. Meanwhile, USA strawberry cultivars were distinguished by their increased presence of two specific ester compounds and the notable γ-decalactone.

Upon clustering using the KVCs, it became evident that Korean cultivars align more closely with Japanese cultivars than with those from the USA, suggesting potential similarities in aromatic profiles or breeding histories between Korea and Japan.

### 3.5 PLS-DA and hierarchical clustering using 14 selected key VOCs

Building on the Key Volatile Compounds (KVC) identified in the prior research by Ulrich et al (Ulrich et al., 2007; Ulrich and Olbricht, 2016) and the VOCs selected based on the Panel Test by Z Fan et al (Fan et al., 2021a), which positively impact consumers, our analysis concentrated on the 14 VOCs that were consistently chosen in both studies. Since the focus was on determining whether these VOCs could effectively differentiate cultivars, we randomly selected 12 cultivars for our study. Utilizing Partial Least Squares Discriminant Analysis (PLS-DA), we analyzed these 14 VOCs and found that the first two components accounted for 40.2% of the total variation in the data. When conducting a PLS-DA analysis using all VOCs collectively, which accounted for 29% of the total variance and demonstrated significant overlap in the 95% confidence regions (**Supplementary Figure S2A**), the cultivars were not effectively separated. In contrast, the PLS-DA based on the selected 14 VOCs indicated more effective differentiation between the cultivars. Following this observation, we further analyzed these 14 VOCs and discovered that the first two components accounted for 40.2% of the total variation in the data, as depicted in **Supplementary Figure S2B**.

To explore the variation trend of relative contents of differential metabolites in different samples, K-means clustering analysis was performed (**Supplementary Figure S3**). The first principal component (PC1) accounted for 23.2% and the second principal component (PC2) accounted for 17.2% of the total variance, collectively explaining 40.4%. In the clustering results, Cluster1 included cultivars such as ‘Chandler’, ‘Diamante’, ‘Kuemsil’, ‘Seolhong’, and ‘Suhong’. This cluster was characterized by a high concentration of aldehyde compounds like nonanal, 2-hexenal (E), 2-pentenal (E), and benzaldehyde. Cluster2, comprised ‘Aiberry’, ‘Daeun’, ‘Dahong’, ‘NS1817002’, ‘Suhyang’, and ‘Sukyung’, was notable for abundant amount of butanoic acid classified in hexyl ester. ‘NS211504’ was independently classified into Cluster3 and exhibited high concentrations of specific Ester compounds such as butanoic acid, butyl ester, Acetic acid, hexyl ester, and Acetic acid, butyl ester. Specific classifications and contents of differential metabolites are shown in **Supplementary Figure S3B**.

### 3.6 Heatmap based on the selected 9 VOCs and lactone compound concentrations

Among the 14 selected VOCs, we applied the method described in Chapter 3.3 to remove compounds with a ω² value less than 0.4. As a result, this led to the exclusion of three Ester compounds and two Aldehyde compounds, leaving nine VOCs identified as valuable Target Compounds. Based on these 9 VOCs, we conducted Hierarchical Clustering on all cultivars and visualized the results through a heatmap (**Supplementary Figure S4**). Each cultivar was color-coded to indicate its country of origin, with shades closer to red indicating a higher relative concentration of the respective compound. Similar to the heatmap generated using only KVCs, USA cultivars clustered closely together, while the oriental cultivars derived from Korea and Japan were clustered more broadly without a specific pattern. Overall, USA cultivars exhibited abundant content of Ester compounds, while Korean and Japanese cultivars exhibited higher proportion of Aldehyde and Ketone compounds.

**Figure 6:**
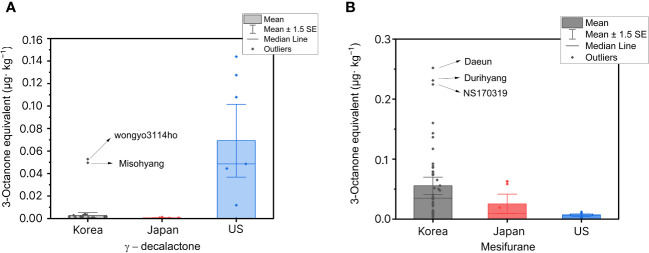
The concentrations of γ-decalactone and mesifurane among strawberry cultivars from Korea, Japan, and selected USA cultivars are represented in terms of their equivalent concentration to the internal standard, 3-Octanone, using Box and Whisker plots. The box illustrates the interquartile range, with the internal line denoting the median. Outliers are shown as distinct points outside the whiskers, and the mean is highlighted.

To gain further insights into the levels of Lactone compounds, we conducted a comparative analysis of their concentrations in different strawberry cultivars from Korea, Japan, and USA (**Figure 6**). The results were visualized using a Box and Whisker plot. For USA cultivars, we specifically selected cultivars known to have high concentrations of certain compounds, including γ-decalactone, to serve as benchmarks for breeding purposes. Consistent with the heatmap findings, the mean and median values of γ-decalactone were higher in USA cultivars. Specifically, ‘Chandler’, and ‘Diamante’, ‘Darselect’ showed high levels of γ-decalactone. Although Korean and Japanese cultivars have lower concentrations compared to their USA counterparts overall, ‘Wongyo3114ho’ and ‘Misohyang’ from Korea exhibited relatively higher levels approaching the median value observed in USA cultivars. In addition to our investigation of γ-decalactone, we turned our attention to mesifurane, another compound of interest. Mesifurane, a lactone-derived volatile, imparts a sweet, caramel-like aroma. Due to its pronounced presence relative to its odor thresholds and its potential as a target compound, mesifurane stands out as a significant player in the aromatic profile of strawberries (Ulrich et al., 1997). Notably, among the cultivars we examined, ‘Daeun’, ‘Durihyang’, and ‘NS170319’ from Korea consistently exhibited elevated concentrations of mesifurane. This highlights the unique aromatic characteristics of these specific Korean strawberries and suggests potential avenues for further exploration and targeted breeding strategies.

## 4 Discussion

### 4.1 Characteristics of fruit flavor from Korean strawberry cultivars and comparisons with foreign cultivars

Our research focused on exploring the unique characteristics of VOCs from most of Korean-derived strawberry cultivars that were recently bred, complemented by few other international cultivars.

The variation of total VOC content in strawberry fruits depended on the cultivars. Among the cultivars, ‘Seolhong,’ had the highest VOC content, exhibiting approximately three times higher than ‘Vitaberry,’ Notably, both ‘Seolhong’ and ‘Vitaberry’ are Korean cultivars. It is also intriguing that ‘Seolhong’ is resistance to anthracnose disease in Korea.

In Korea, strawberry cultivars were developed through crossbreeding among a limited selection of parent cultivars and their seedlings, which indicates that the genetic diversity may be very narrow. However, the variations in VOC content among these Korean cultivars suggest the potential for enhancing aroma profiles through selective breeding and improved efforts.

In our study, there was no significant variation in the total number of VOCs among strawberry cultivars tested. It is preferable to focus on the compositional differences rather than the quantity of compounds. Interestingly, Japanese cultivars, according to market share data from 2015, exhibited as ‘Tochiotome’ (48%), ‘Amaou’ (19%), and ‘Benihoppe’ (9%) (Nobuko, 2015), while the number of VOCs in these cultivars followed a similar order (‘Tochiotome’ having 93, ‘Amaou’ having 91, and ‘Benihoppe’ having 90 different compounds). Particularly, ‘Benihoppe’ did not contain isopentyl alcohol (1-butanol, 3-methyl-) in any of our samples, while this compound is known for its antifungal activity against plant diseases (Thangaraj et al., 2023). The market share of cultivars reflects not only consumer preferences but also factors such as cultivation simplicity. The presence or absence of key compounds could significantly impact market success. Notably, the premium strawberry cultivar ‘Jukhyang,’ known for its high quality, also displayed a substantial number of aromatic compounds (96 compounds), which suggests that this might not be a mere coincidence.

Given the extensive cultivar of VOCs, it may be more efficient to concentrate breeding efforts on a select few compounds of significant importance. For instance, research groups working on the enhancement of strawberry flavors have specifically aimed to increase the concentrations of γ-decalactone, mesifurane, and methyl anthranilate, considering the effectiveness and genetic characteristics of these compounds (Chambers et al., 2014; Sánchez-Sevilla et al., 2014; Pillet et al., 2017; Barbey et al., 2021; Fan et al., 2022). The *FaFAD1* gene, known to impact the production of γ-decalactone and situated at a distinct genetic locus, emerges as a promising target for marker-assisted seedling selection (Sánchez-Sevilla et al., 2014; Oh et al., 2021). The ‘Misohyang’ cultivar, a product of the 2009 crossbreeding between ‘Darselect’ and ‘Wongyo3111ho’, has been previously identified to possess the *FaFAD1* gene (Sun Yi et al., 2022). Our study reinforces this, highlighting the notably elevated VOCs levels in ‘Misohyang’ relative to other Korean cultivars. For countries like Korea, the private breeding industry joined at a later stage, and strategically incorporating these specific compounds into Korean cultivars could provide multiple benefits. This includes aligning with consumer preferences and optimizing cultivation techniques.

### 4.2 Strategic enhancement of strawberry cultivars: focused breeding for improved VOC profiles and consumer preference alignment

In selecting a target compound for cultivar enhancement, one must consider various factors, especially the ability of the added fragrance to harmonize with existing aromas and appeal to consumer preferences. Identifying compounds with high heritability is crucial for establishing a breeding system resilient to diverse environmental conditions with high levels of disease resistance. Moreover, comparing the lack of specific VOCs in certain groups of cultivars enables targeted efforts to improve their concentration. Molecular-assisted breeding also facilitate identifying loci and genetic markers associated with the production of VOCs. In this study, screening VOCs from 61 strawberry cultivars from Korea, USA, Japan, and France provided 122 detectable VOCs.

Among these identified 122 VOCs, hexanal emerged as the compound with the highest average concentration in Korean samples. However, despite its prevalence, the ANOVA results revealed a ω² value of less than 0.4 (specifically, 0.396). This suggests that accumulation of hexanal is influenced more by environmental factors than genetic factors. The 2-hexenal, (E)- ranked as the third most abundant compound in Korean samples. Notably, its concentration in Korean cultivars was approximately two times higher than in USA cultivars. Previous consumer preference tests have indicated that this compound might have a negative impact on consumer perception, suggesting that future breeding strategies should aim to reduce its concentration progressively. Among the ester compounds, butanoic acid and methyl ester were the most frequently detected compounds. Given its fruity and sweet aroma, the compounds hold potential target for future breeding. In the terpene category, the compound detected at the highest concentration was linalool, which has a lavender-like aroma. ANOVA results indicated that it possesses high heritability. Interestingly, Korean cultivars had a notably higher concentration of linalool than USA cultivars, suggesting its significant contribution to the unique aroma of Korean strawberries. Furthermore, consumer tests have shown its positive impact on aroma perception. In the ketone category, methyl isobutyl ketone was the most abundant compound. Known for its camphor like odor, it can be unpleasant at high concentrations. This compound was influenced significantly by genotype and was detected at higher levels in Korean cultivars compared to USA cultivars. Reducing its concentration should be important in future breeding efforts.

The γ-decalactone and γ-dodecalactone, both lactones known for inducing a sweet and peach-like aroma, have been recognized as significant contributors to consumer preference in flavor profiles (Ulrich and Olbricht, 2016; Fan et al., 2021a). A previous study has calculated a Pearson correlation coefficient exceeding 0.6 between these two volatile organic compounds (VOCs), indicating a strong positive correlation (Ulrich and Olbricht, 2016; Rey-Serra et al., 2022). This supports the hypothesis that both compounds share a common biosynthetic route via the beta-oxidation pathway of fatty acids in yeast (An et al., 2013; Yu et al., 2023). Furthermore, γ-decalactone is not present without the expression of the omega-6 fatty acid desaturase gene, *FaFAD1*, supporting this metabolic pathway (Oh et al., 2021). Although our research did not demonstrate a clear correlation between γ-decalactone and γ-dodecalactone, the extensive studies conducted on these lactones suggest that they could be leveraged for effective breeding practices. Given the potential of γ-decalactone to positively influence consumer preference and its currently low levels in Korean strawberry cultivars, it will be advantageous to select γ-decalactone as a target compound for breeding new cultivars.

### 4.3 Optimizing strawberry aroma through genetic breeding: targeting γ-decalactone in cultivar development based on comprehensive VOC analysis

**Figure 7:**
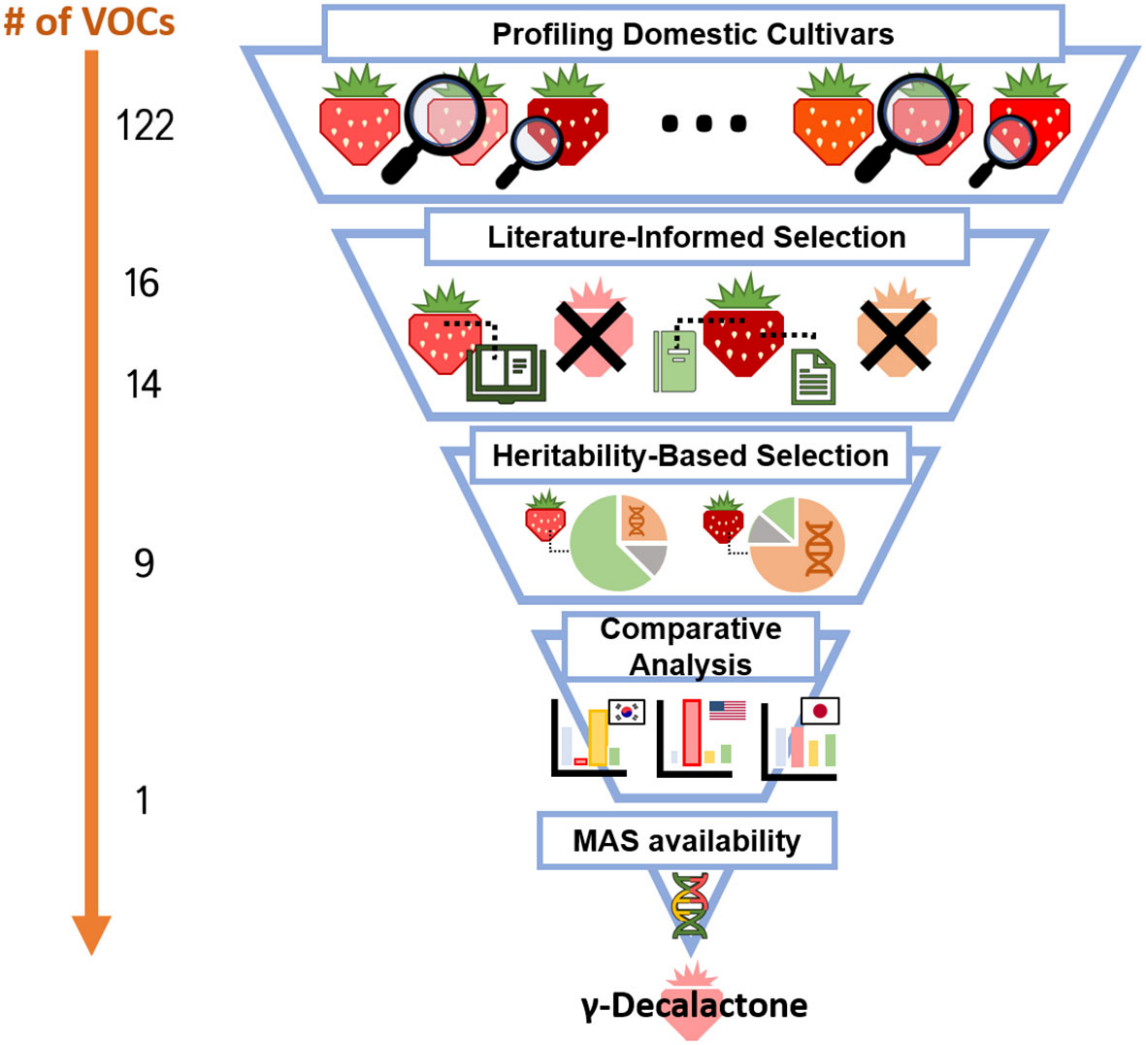
Strategic selection of target VOCs for enhanced strawberry breeding. The steps involve: 1) Profiling VOCs in the various strawberry cultivars; 2) Identifying VOCs highly correlated with flavor and consumer preferences through literature review; 3) Selecting compounds influenced predominantly by genetic factors rather than environmental conditions; 4) Conducting a cross-national comparison to pinpoint areas for improvement in domestic cultivars; 5) Upon satisfying the aforementioned criteria, the compound suitable for making genetic marker is selected as the target compound. γ-Decalactone is exemplified.

In our study, the process of determining the target compound is summarized in **Figure 7**. Our analysis of 61 strawberry cultivars from Korea and other countries led to the detection of 122 VOCs. Among the detected compounds, 14 VOCs known to significantly influence strawberry aroma and consumer preference were selected, and the one-way ANOVA test supported that nine of these were confirmed to have high heritability. Among the nine compounds, γ-decalactone stands out for its significant impact on aroma. Because of the genetic and genomic information for the γ-decalactone metabolic mechanism is well characterized, improving this fruity aroma flavor will provide great benefits to Korean strawberries. Additionally, developing a new cultivar such as ‘Misohyang’ with a relatively high concentration of γ-decalactone would be important for future breeding, but multiple disease resistance should be combined together.

In the process of interpreting the aroma profile, various points must be considered. For example, although alkanes constituted a substantial portion of the total VOCs, they were excluded due to their minimal impact on the overall aroma profile (Ouellette and Rawn, 2015). On the other hand, the quantity of VOCs classified in alcohols and aldehydes correlated with flavors in strawberries, while terpenes exerted a significant impact on sensory evaluation despite relatively low content (Schieberle, 1994; Pesis, 2005; Porter et al., 2023). Given the importance of compounds including γ-decalactone and mesifurane which are classified in lactone and ketone groups, a comparison analysis was also performed between these two groups in this study (Abouelenein et al., 2023).

Furthermore, it is worth noting that in this experiment, TD tubes with Tenax TA resin as the adsorbent were employed instead of PDMS materials like SPME or HiSorb. However, the use of TD tubes with Tenax/Carbograph resin has been reported to have higher adsorption rates for alcohols and aldehydes but lower sensitivity in capturing furans and lactones, compared to other methods (Cheng et al., 2021). Given this, for future studies particularly focused on lactone compounds, it might be beneficial to adopt a VOC sorption method that employs PDMS materials.

Moreover, indiscriminate augmentation of compound levels may not always be advisable. While research predominantly highlights the positive effects of linalool, there are reports suggesting that it can enhance ‘green’ flavor notes (Fan et al., 2021b). Hexanoic acid, on the other hand, is known to associate with sweaty or cheesy off-flavors (Du et al., 2011), although conflicting results also suggest potential benefits for sweetness and aroma enhancement (Pang et al., 2021). It would be essential to conduct more meticulously designed field experiments focused on flavor and determine the optimal levels while balancing and accessing the influence on other aroma compounds during fruit ripening and postharvest stages.

### 4.4 Crafting the future of strawberries: enhancing aroma through innovative breeding techniques

In the case of tomatoes, breeding programs have predominantly focused on improving yield, disease resistance, and shelf-life, a strategy that has inadvertently led to a decline in their intrinsic flavor, a trend increasingly met with consumer dissatisfaction (Klee, 2010). Similarly, initial breeding programs for strawberries also concentrated on enhancing traits like yield and disease resistance. This focus has led to the disappearance of certain genes related to flavor compounds in cultivated strawberries (Aharoni et al., 2004). While the specific history of breeding related to strawberry aroma is not well-documented, both strawberries and tomatoes share similar consumer preferences and biosynthetic pathways (Klee and Tieman, 2018), suggesting a parallel situation in terms of flavor development in strawberries. Interestingly, the rich grape-like aromatic compound methyl anthranilate, abundantly present in wild-type strawberries, is either absent or found in trace amounts in most commercial cultivars (Urrutia et al., 2017). It suggests that numerous flavors from wild strawberries have not yet been introduced into modern strawberry cultivars. Recent efforts have been made to enhance sweetness and fruity aroma of cultivated strawberries; however, this endeavor faces challenges. Due to the genome complexity of octoploid strawberry, control of inheritable traits such as flavor is difficult process through the traditional breeding method. Furthermore, it’s only in recent times that there has been a rapid increase in research focusing on the compounds related to strawberry aroma and their associated metabolic information (Ulrich et al., 2018). Currently, in most countries including Korea, the breeding of strawberry cultivars is predominantly carried out using traditional breeding methods, which could require at least four years to develop new cultivars and would not be easy procedure to combine genetically diverse traits together into a single cultivar (Kang Hee et al., 2007; Kim et al., 2019; Park, 2021; Zurn et al., 2022).

**Figure 8:**
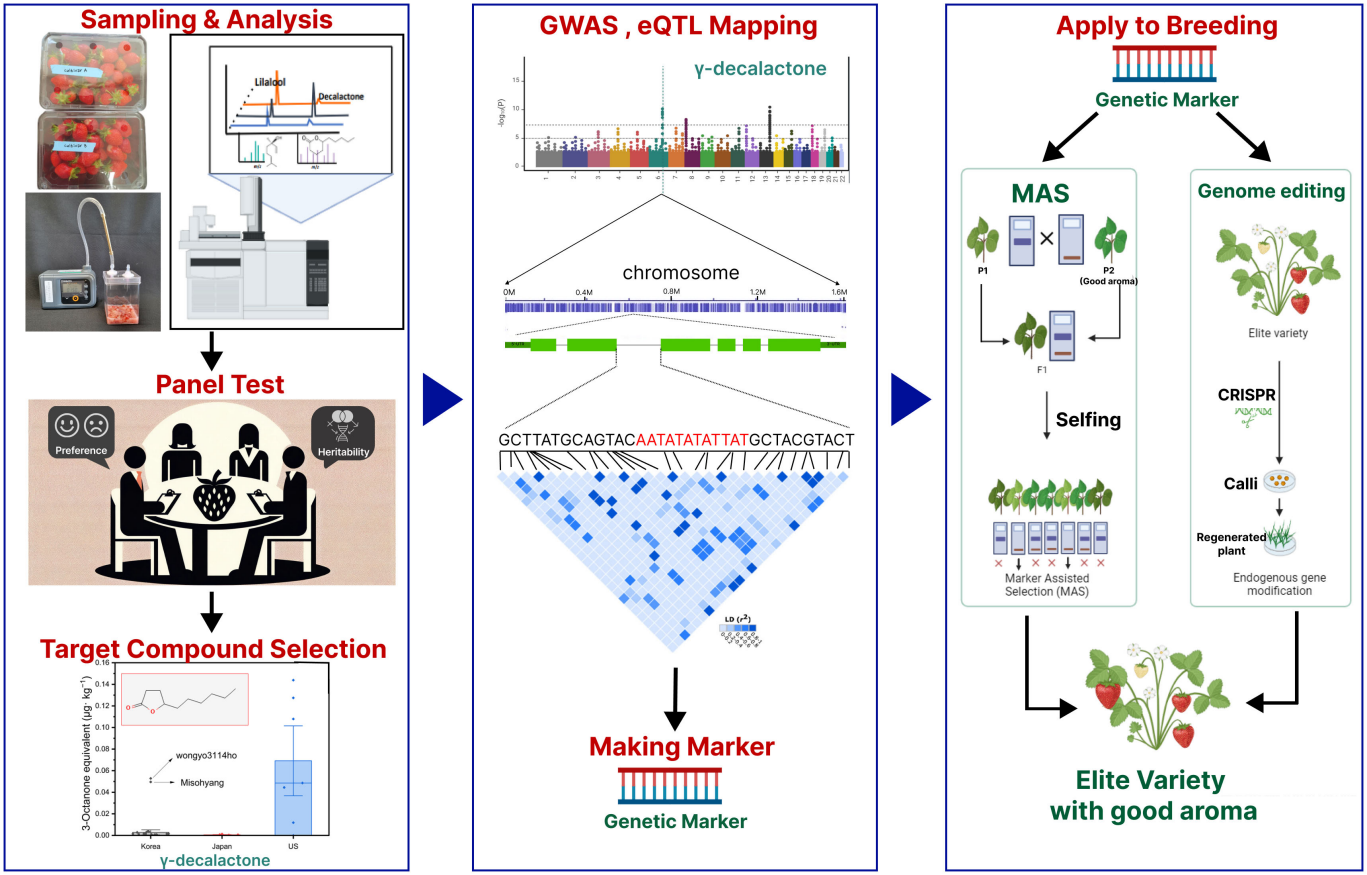
Scheme for a breeding program to enhance strawberry aroma using DNA markers.

Using molecular breeding tools and advanced genomic resources, we can effectively discover any desirable traits and accelerate the overall breeding cycle for the development of new cultivar. As shown in **Figure 8**, future strawberries can be enhanced through genomics-assisted breeding approaches. To effectively implement DNA marker-assisted breeding, the initial step is to establish a target compound that enhances the desired aroma. This process begins with the collection and analysis of plant samples, followed by a panel test to determine consumer preferences and identify compounds with high heritability for the efficient progression of the breeding program.

Breeding programs utilizing genetic markers must prioritize clear objectives for cultivar development. If the goal is to enhance the aroma of strawberries, it is crucial to accurately determine which scent characteristics to improve by setting the target compounds. Successful development of genetic markers hinges on this selection. This is because in most crops, a considerable number of VOCs share the same biochemical synthetic pathways, meaning that enhancing one compound could affect the overall VOCs profile and other traits (Eduardo et al., 2013). Once the target compound is determined, GWAS and eQTL mapping studies are employed to locate genomic regions affecting the concentration of target compounds. Genetic markers developed from loci associated with desired traits can be used in marker-assisted selection (MAS) to enhance the aroma profiles of strawberries. Moreover, integrating genome editing techniques like CRISPR with MAS offers a promising method for accelerating crop variety improvement (Nadeem et al., 2018). This synergy significantly boosts efficiency and precision in crop improvement by allowing the direct alteration of less favorable alleles into more advantageous ones, thus accelerating the breeding process. Such an approach is especially beneficial in the development of strawberry cultivars with enhanced aroma profiles, combining the strengths of both MAS and genome editing for a more rapid and targeted improvement. Of course, aside from MAS and gene editing techniques for cultivar improvement, there are other various methods that could be used to enhance fruity aroma in strawberries. For instance, a study in the National Institute of Vegetable and Tea Science in Japan has shown the development of strawberry cultivars through the technique of polyploidization. While the initial decaploid strawberries demonstrated a strong fragrance, they suffered from poor fruit quality. However, through meticulous selection, the decaploid cultivar ‘Tokun’ was developed, which has unique aromas like caramel, coconut, and peach (Morishita, 1994; Noguchi, 2011). This underscores that the polyploidization method could be instrumental in modifying the aroma profile of octoploid strawberries. In the face of challenges due to limited genetic diversity, exploring such alternative breeding methods might be crucial. Even in such instances, it will be important to identify target compounds to enhance specific fragrance components of strawberry fruits. As we look towards the future, our comprehensive database developed from this study will be invaluable in Korean breeding programs aimed at enhancing strawberry flavor.

## Data availability statement

The original contributions presented in the study are included in the article/**Supplementary Material**. Further inquiries can be directed to the corresponding author.

## Author contributions

EJ: Writing – original draft, Data curation, Formal analysis, Investigation, Validation, Visualization. ED: Writing – original draft, Data curation, Investigation, Visualization. CG: Writing – review & editing, Investigation, Resources. SK: Conceptualization, Investigation, Resources, Writing – original draft. SYL: Conceptualization, Investigation, Resources, Writing – original draft. SL: Funding acquisition, Supervision, Validation, Writing – review & editing. KK: Funding acquisition, Supervision, Validation, Writing – review & editing, Conceptualization, Project administration.

## Supplementary material

Supplementary Figure 1 Score plot of the Principal Component Analysis results for the total VOCs of 61 strawberry cultivars

Supplementary Table 1 Information About 122 VOCs Compounds Analyzed and results of the heritability analysis of all detected VOC concentrations
