# Supplementary figures and images for: Analysis of volatile organic compounds in Korean-bred strawberries: insights for improving fruit flavor
Source: Front Plant Sci. 2024 Mar 18;15:1360050. doi: 10.3389/fpls.2024.1360050 (PMC10982345; doi:10.3389/fpls.2024.1360050)

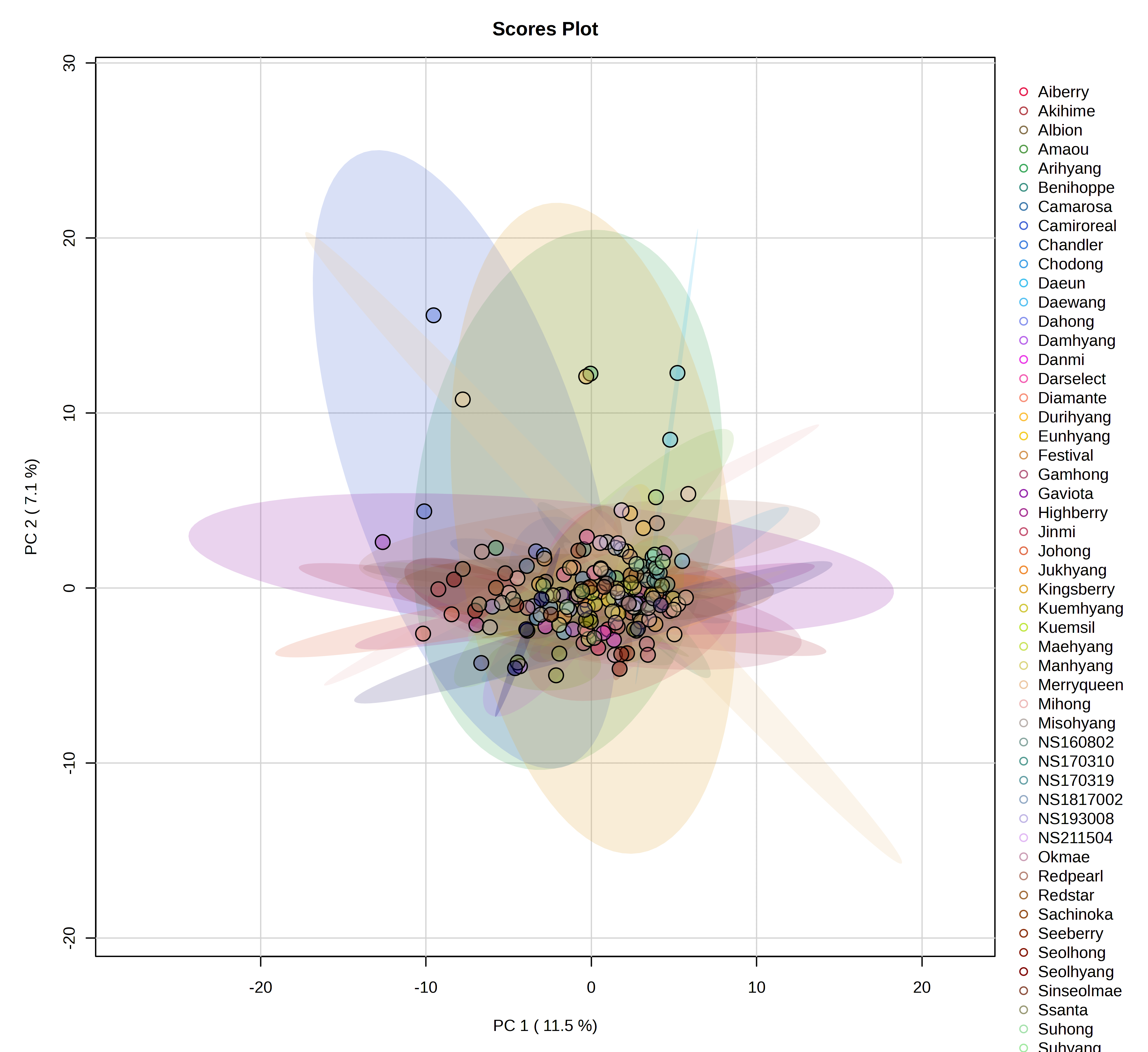

Supplement: Supplementary Figure 1 — Score plot of the Principal Component Analysis results for the total VOCs of 61 strawberry cultivars [file Image_1.tif]

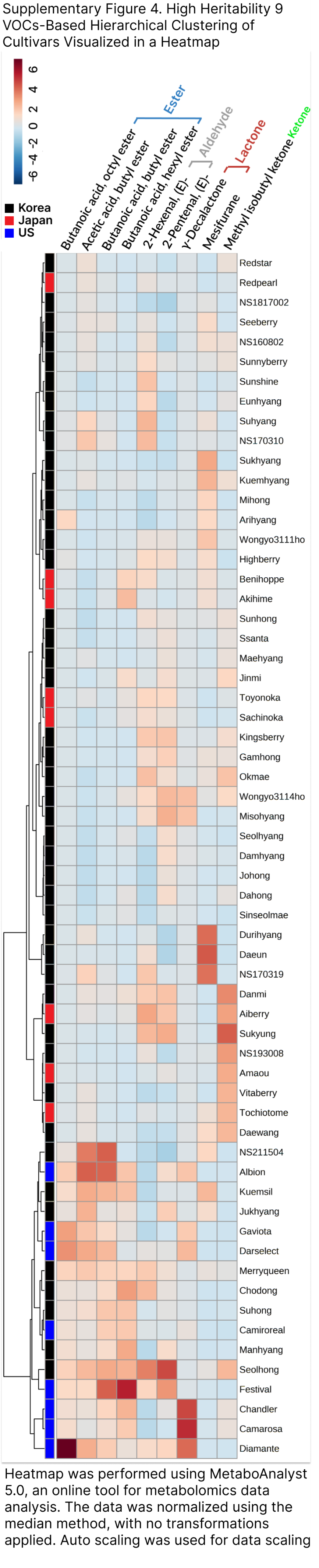

Supplement: Supplementary file 4 [file Image_4.tiff]
